# Examining clinical pharmacist interventions and identifying opioid medication-related issues in patients with cancer

**DOI:** 10.1177/10781552241279027

**Published:** 2024-08-28

**Authors:** Amjad Anwar, Nirmal Malik, Adeel Siddiqui, Sunil Shrestha, Omar Akhlaq Bhutta, Saba Mazhar, Muhammad Rehan Khan, Hafiz Muhammad Usman

**Affiliations:** 1 Department of Pharmacy, 66799 Shaukat Khanum Memorial Cancer Hospital & Research Centre, Lahore, Pakistan; 2 School of Pharmacy, 65210 Monash University Malaysia, Subang Jaya, Malaysia; 3 Department of Research and Academics, Kathmandu Cancer Center, Tathali, Nepal

**Keywords:** Clinical pharmacist, clinical pharmacist interventions, opioid medications, medication-related issue, patients with cancer, patient safety

## Abstract

**Introduction:**

Opioid medications are crucial for managing pain among patients with cancer. Yet, inappropriate prescribing and medication issues can compromise patient safety and quality of care. Clinical pharmacists play a significant role in optimizing opioid therapy and addressing issues related to opioid medication use.

**Objectives:**

This study aimed to examine clinical pharmacist interventions and identify opioid medication-related issues in patients with cancer.

**Method:**

We conducted a retrospective observational study at Shaukat Khanum Memorial Cancer Hospital and Research Center in Lahore, Pakistan, conducting a chart review from 1^st^ July 2021 to 31^st^ December 2021.

**Results:**

Out of 10,534 opioid medication orders, we documented a total of 974 interventions based on our inclusion criteria. Tramadol and morphine accounted for most of these interventions, comprising 49.27% (n = 475) and 40.04% (n = 386), respectively. Regarding clinical significance, 41.70% (n = 406) were deemed significant, while 37.36% (n = 365) were somewhat significant. The majority of interventions, i.e., 54.05% (n = 521), primarily aimed at optimizing patient outcomes, followed by a secondary aim of improvements in communication, i.e., 25.52% (n = 246).

**Conclusion:**

This study establishes the evaluation of clinical pharmacist interventions on opioid medication use in patients with cancer, an issue particularly in oncology settings in Pakistan. The findings emphasize the crucial role of clinical pharmacists in addressing issues related to opioid issue medications, thus improving patient safety and optimizing opioid use for patient well-being.

## Introduction

Cancer remains a significant health challenge across Asia, accounting for as much as 49.3% of global cancer cases.^1^ Unfortunately, a substantial portion of cancer diagnoses occur at advanced stages, often accompanied by severe pain.^2^ Pain was prevalent in 44.5% of the cases, with 30.6% of patients experiencing moderate to severe levels.^3^ Addressing this issue is critical, as inadequate pain management can adversely affect multiple facets of a patient's well-being.^4–7^

Efforts to manage cancer pain have been guided by the World Health Organization's (WHO) analgesic ladder, which advocates for the use of opioid analgesics for moderate to severe pain.^8,9^ While opioids are effective, their use also raises safety concerns due to potential adverse effects and the risk of opioid use disorders.^10,11^ Furthermore, the escalating harms associated with prescription opioids have become a worrisome issue, particularly in lower and middle-income countries.^12^ Challenges in opioid medication management can be compounded by prescribing issues, which may lead issue to adverse patient outcomes.^13^ The increase in opioid-related harms emphasizes the necessity for an effective Opioid Stewardship Program (OSP) aimed at regulating opioid prescription practices.^14,15^ Opioid stewardship aims to balance the therapeutic benefits of opioids with minimizing risks and preventing misuse.^14^ This context highlights the importance of clinical pharmacists, who are crucial in optimizing drug therapy and reducing adverse events.^16–19^

Our study at Shaukat Khanum Memorial Cancer Hospital & Research Centre in Lahore aims to address the gap in research on opioid stewardship in Pakistan by evaluating the impact of clinical pharmacist interventions on improving opioid prescribing practices. Our study at Shaukat Khanum Memorial Cancer Hospital & Research Centre in Lahore aims to address the gap in research on opioid stewardship in Pakistan by evaluating the impact of clinical pharmacist interventions on improving opioid prescribing practices. Clinical pharmacists at our institution play a crucial role in ensuring the safe and effective use of opioids in patients with cancer. Their responsibilities include addressing physician queries, developing patient care plans, discussing recommendations, and mitigating adverse drug events (ADEs) related to opioid toxicity. We aim to assess the acceptance and efficacy of these interventions in optimizing opioid prescribing practices in patients with cancer.

## Methodology

### Design and setting

This was a retrospective observational study based on the data taken from a hospital that extends its comprehensive oncology services to patients residing in Lahore and hailing from various parts of the nation, in addition to neighboring areas such as Afghanistan.^20^

### Study period

The study cover**s** a period of 6 months, starting from 1^st^ July 2021 to 31^st^ December 2021.

### Ethical approval

Research Ethics Committee and institutional review board (IRB) at Shaukat Khanum Memorial Cancer Hospital and Research Center has reviewed to grant the exempt status and waiver of informed consent to this retrospective research study. This was a research project of the resident pharmacist. The study was then conducted by the hospital's clinical pharmacists, encompassing resident pharmacists, staff pharmacists, and assistant managers from the clinical pharmacy services. The research adhered diligently to the established protocol and all pertinent guidelines and regulations.

### Inclusion / exclusion criteria

The study included only patients with cancer who were prescribed opioid medications to manage their pain and had opioid orders initiated by a pharmacist, indicating that the pharmacist recommended starting this therapy. This included both adult and pediatric cases. These patients were admitted under oncology, palliative, or surgical services within the designated study period. Opioid orders prescribed for patients in the ambulatory setting are excluded. Furthermore, the study only included opioid prescription interventions for patients with cancer and omitted patients who were admitted with any other conditions. Similarly, unrecorded verbal interventions discussed during clinical rounds and unanswered interventions were excluded from the study.

### Study outcomes

The primary outcome of the study was to evaluate the number of documented clinical pharmacist interventions related to opioid prescribing practice issues and the acceptance rate of these interventions. The secondary outcome was to assess the specific opioids involved in the interventions, the clinical significance of the interventions, and the number and type of interventions conducted.

### Intervention documentation and data collection

The electronic hospital information system (HIS) has a built-in special intervention form. It includes the physician's prescribed order, the order suggested by the pharmacist, the severity of the intervention, rationale, recommendation, and outcome. The pharmacist can also enter free text documentation to explain and provide further information regarding clarifying the intervention. The HIS's pharmacist intervention form also includes a physician's decision and response about the pharmacist's intervention. The clinical decision support system (CDSS) aids pharmacists in preventing prescription issues by issuing alerts for deviations from drug dosage limits, drug interactions, high-alert medications, duplicate drug orders, duplicate therapies, drug allergies, and patient weight and height variations recorded in the patient's file. Additionally, the department monitors clinical pharmacist activities, such as interventions and reporting adverse drug reactions (ADRs), as part of its key performance indicators. High-alert medications undergo double-checks for added safety measures.

Clinical pharmacists recorded their interventions for opioid orders during the prescription appropriateness evaluation by selecting the relevant type of intervention from a list of different types. With the cooperation of the hospital's Information Technology department and electronic medical record, all documented interventions for opioid medicines in the system that met the criteria during the set period were pin-pointed. Patients who met the inclusion criteria were located, and the report was extracted. The type of intervention includes drug allergy, drug-drug interaction, dosage calculation, inappropriate duration, inappropriate route, inappropriate dose, inappropriate schedule, inappropriate rate of infusion, a dosage type issue, double entry, contraindication, drug-nutrient interaction, wrong indication, therapeutic duplication, drug-disease interaction, IV to PO switch, medication mismatch, look-alike, sound-alike issue, ADRs, missing instructions, non-formulary order, better therapeutic choice, short expiry, medication reconciliation, and other interventions. The clinical significance of pharmacist's interventions was modified, as shown in Table 1, along with the specialized clinical judgment of the clinical pharmacist reviewing the interventions.^21^ The clinical significance assessment was done considering the impression of suggestions for prescribed orders or modifications and adjustments in treatment for patient care, communication, and safety of the drug used. Similarly, the intervention outcome as used by Al-Quteimat et al. was modified to assess the outcomes by a clinical pharmacist to review the outcomes as shown in Table 2.^22^

**Table 1.** Description of tool for clinical significance of interventions.

1	Very Significant	The recommendation prevents real or potential damage to a vital organ from a drug interaction or contraindication.
2	Significant	The recommendation is therapeutic and improves the patient's quality of life.
3	Somewhat Significant	The recommendation is preventive and improves the patient's condition to some degree according to the hospital's standard policy.
4	Insignificant	For informative purposes only - general recommendations, not specific to a particular patient.
5	Sub-optimal Intervention	Inappropriate recommendations could lead to a worsening of the patient's condition.

**Table 2.** Description of tool for intervention outcomes.

1	Optimize Therapy	Related to medication reconciliations and correlation of physician orders.
2	Avoided ADRs	Related to prescribing errors such as doses exceeding two times the patient's weight, very high doses, drug-drug interactions, or inappropriate renal/hepatic adjustments.
3	Improved Communication	Involves clarifying missing information, particularly for weight-based doses or prn (as needed) orders for breakthrough pain, following pain management guidelines.
4	Cost Saving	Related to dosage form conversion and cost-effective intervention
5	Sub-optimal Therapy	Involves interventions that do not align with promoting optimal prescribing practices.

prn = ‘pro re nata’ i.e., as needed.

### Data analysis

The interventions were examined for the most frequently encountered opioid drugs, the type and number of each included intervention, and their clinical importance. The physicians’ acceptance rate was calculated using simple descriptive statistical calculations for percentages.

## Results

**Figure 1.**
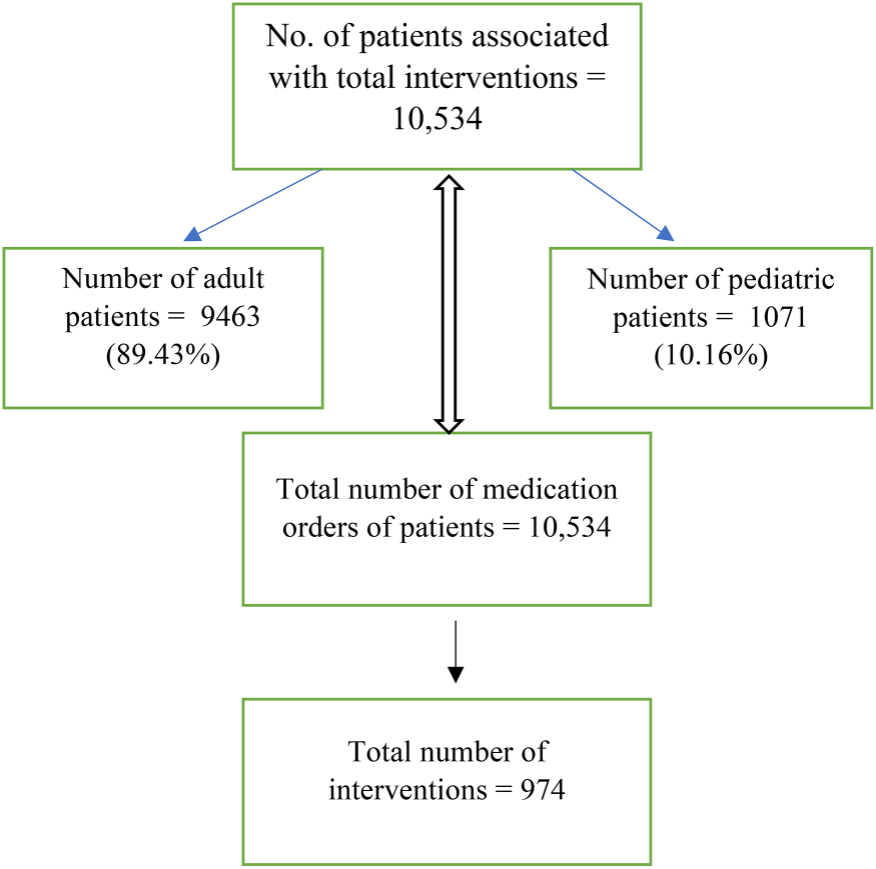
Flowchart indicating the total number of patients, total number of opioid medication orders and total number of interventions.

Figure 1 shows the flowchart presenting an overview of the study results related to the number of patients, opioid medication orders, and interventions. The total number of patients involved in the study is 10,534. Of these, 9463 are adult patients, constituting 89.43% of the total patient population, while 1071 are pediatric patients, making up 10.16%. The total number of medication orders placed by these patients is also 10,534, which matches the number of patients, suggesting that each patient had one medication order associated with them. Finally, the total number of interventions in the study is 974.

Table 3 presents the socio-demographic characteristics and disease information of the patients with cancer. A total of 10,534 prescriptions were analyzed, involving 9463 medication orders for adult patients, of which 32.27% were medication orders for males and 57.55% were for females, with a median age of 45. Additionally, there were 1071 medication orders for pediatric patients, comprising 6.22% for males and 3.94% for females, with a median age of 9.5 years. Among the pharmacist interventions for patients with cancer, diagnoses included 37 different types of cancer, each at varying stages: 0 (0.62%), 1 (16.38%), 2 (32.30%), 3 (27.58%), and 4 (16.08%). Additionally, there were cases classified as binet (0.098%), chronic (2.686%), unstageable (3.899%), stage not applicable (0.327%), and 1006 entries were blank, therefore excluded from the data of type of cancer diagnosed.

**Table 3.** Socio-demographic characteristics and disease information of the patients.

	Number	Percentage
**Adults**		
Median Age	45 years	
Male	3400	32.27
Female	6063	57.55
**Pediatric patients**		
Median Age	9.5 years	
Male	656	6.22
Female	415	3.94
**Type of cancer diagnosed**	37	
**Stages of Cancer**		
0	59	0.62
I	1561	16.38
II	3078	32.30
III	2629	27.58
IV	1533	16.08
Binet	9	0.09
Chronic	256	2.68
Unstageable	372	3.89
Stage not applicable	31	0.32

Table 4 summarizes the total number of interventions for various medications and their acceptance or rejection by physicians. The data reveals that among the 974 total interventions, 898 were accepted, while 76 were rejected. Buprenorphine had 5 interventions, with 3 accepted and 2 rejected. Codeine Phosphate had a higher acceptance rate, with 34 out of 36 interventions accepted. Fentanyl Citrate showed a perfect acceptance record, with all 11 interventions accepted. Morphine Sulphate, which had the highest number of interventions at 384, had 350 accepted and 34 rejected. Pethidine had only 1 intervention, which was rejected. Tapentadol had 56 interventions, with 48 accepted and 8 rejected, while Tramadol HCl, with the highest number of interventions at 481, saw 452 accepted and 29 rejected.

**Table 4.** Total number of interventions and those accepted or rejected by the physicians.

Medications	Total number interventions	Accepted Interventions	Rejected interventions
Buprenorphine	5	3	2
Codeine Phosphate	36	34	2
Fentanyl Citrate	11	11	0
Morphine Sulphate	384	350	34
Pethidine	1	0	1
Tapentadol	56	48	8
Tramadol HCl	481	452	29
	974	898	76

**Figure 2.**
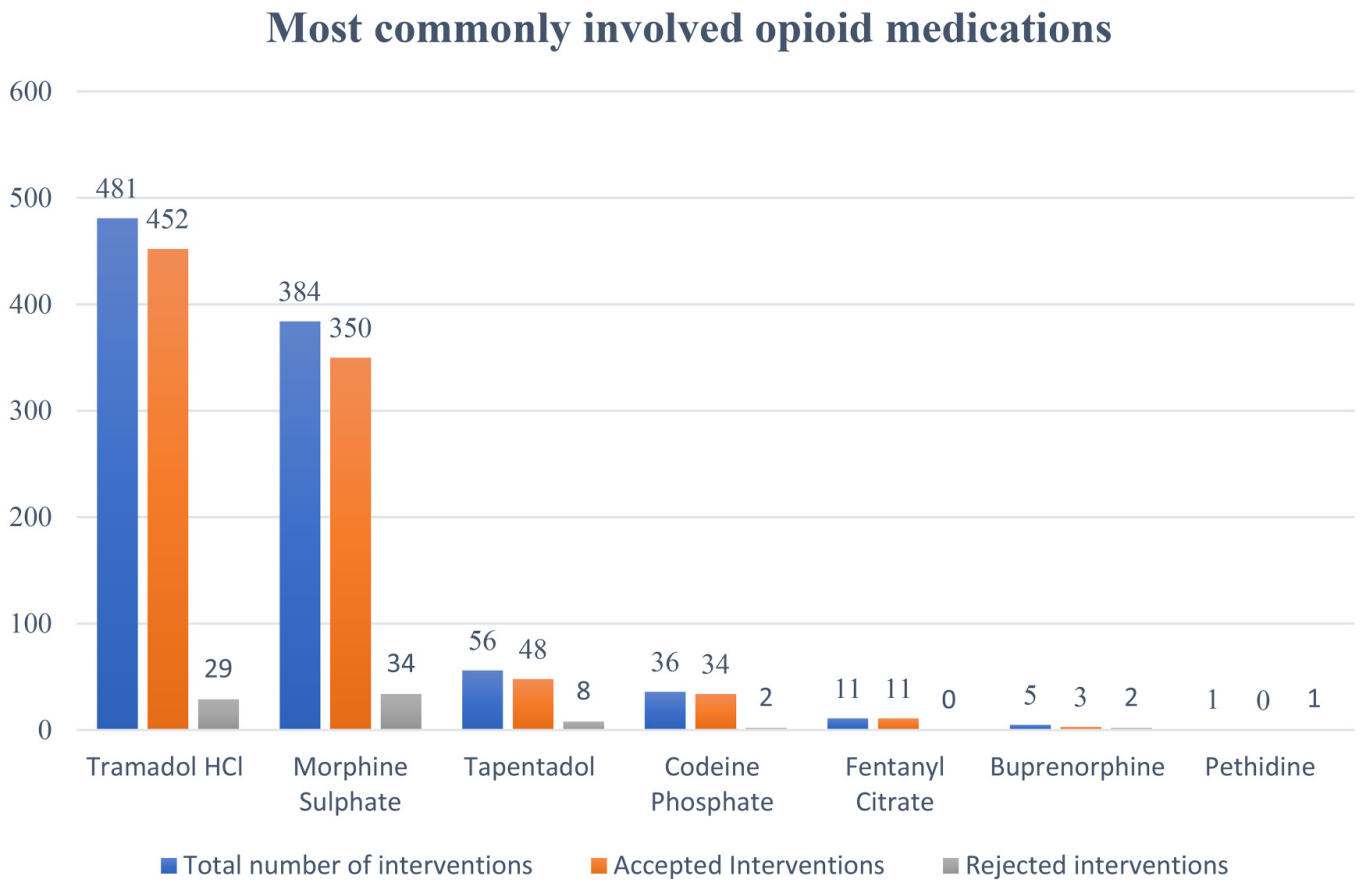
Most commonly involved opioid medications in interventions.

Out of 10,534 opioid medication orders, the total number of clinical pharmacist interventions against opioid prescription included in the analysis was 974. A summary of the number of totals, accepted and rejected interventions on different opioids is given in Figure 2.

Regarding the drug, tramadol was the drug associated with the highest number of interventions, followed by morphine, constituting 49.38% (n = 481) and 39.42% (n = 384) of the total interventions, respectively. Of these 974 documented interventions, 898 were accepted by the doctors, accounting for an acceptance rate of 92.2%.

Most of the clinical pharmacists’ interventions were significant and somewhat significant. The distribution of the clinical significance of the interventions is shown in **Table 5**.

**Table 5.** Clinical significance of clinical pharmacist interventions.

Clinical Significance	Number and Percentage
Very significant	152 (15.66%)
Significant	406 (41.70%)
Somewhat significant	365 (37.36%)
Insignificant	45 (4.66%)
Suboptimal interventions	6 (0.62%)

**Figure 3.**
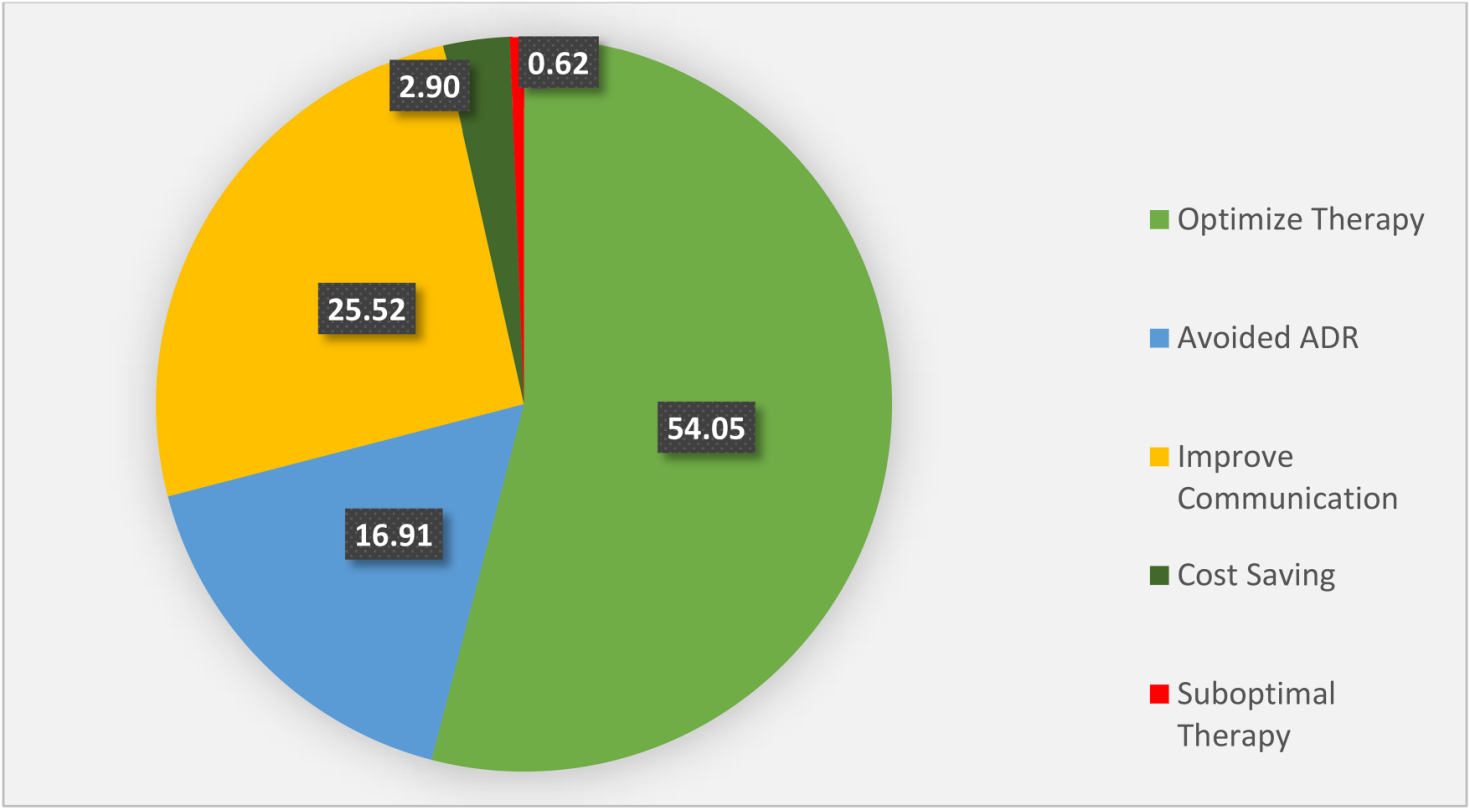
Illustration of clinical pharmacist intervention outcomes (values in percentages).

The prominent intervention outcomes made on opioid prescriptions included optimized therapy, improved communication, avoided ADEs and cost savings. The interventions under optimized therapy included correcting wrong doses, inappropriate schedules, and drug-disease interactions (i.e., 28.02%, 15.35%, and 10.68%, respectively). The data on intervention outcomes is illustrated in Figure 3.

## Discussion

The study highlight**s** the critical role of clinical pharmacists in mitigating and preventing opioids related issues, particularly in oncology settings in Pakistan. Our study demonstrates that clinical pharmacists, integral members of interdisciplinary oncology teams, recommend a significant number of accepted interventions to address diverse drug-related issues, significantly enhancing healthcare provision and ensuring the safe use of opioids for patients. Clinical pharmacists play an important role in patient care, evident from documented interventions in opioid-related prescribing issues and the relevance of such interventions, underscoring the importance of their role in managing opioid risks.^23^ In our study, the intervention rate was 9.24%, consistent with rates ranging from 0.5 to 10.2% reported in the literature for pharmacist interventions in prescription issues.^24–26^ These rates vary widely, with some studies reporting as low as 0.78% and 0.5% interventions in thousands of prescriptions,^27,28^ while others document rates as high as 9.22% in over 14,000 prescriptions.^29^ According to our study, tramadol had the highest percentage of clinical pharmacist interventions (49.38%), followed by morphine (39.43%). The total interventions made for optimizing therapy, i.e., 54.05%, included the ones done for wrong doses (28.02%), inappropriate schedule (15.35%) and drug-disease interactions (10.68%).

The interventions conducted in the study played a significant role in preventing multiple ADEs.^30,31^ These interventions helped mitigate risks such as dose accumulation at high levels, drug-drug interactions, and dosage-induced toxicities. Patients with renal or hepatic impairments required dose modifications to avoid potential complications.^32–36^ The high number of interventions for opioid medications demonstrates the development and application of recommendations to ensure their safe and effective use. This highlights the essential role of clinical pharmacists in optimizing and managing opioid prescriptions, which can help to reduce overuse, conserve resources, and avoid associated adverse responses.^37^

In our study, 92.19% (n = 898/974) of clinical pharmacist interventions were accepted by doctors. This acceptance rate significantly surpasses the 53% reported in a published study,^38^ and exceeds previously reported pharmacist intervention acceptance rates.^24,39^ The precise assessment of opioid prescribing issues and associated underlying opioid-related problems by our clinical pharmacists may help to explain the greater acceptance rate in our study. Disparities in communication strategies for prescription issues, clinical pharmacists’ involvement in daily ward rounds, physicians’ attitudes toward pharmacists, and systems for identifying medication issues may have influenced these acceptance rates across studies.^40^

The results indicate that the majority of interventions focus on optimizing therapies, with over 54% targeting improvements in therapeutic choices, including dose, timing, frequency, dosage form, and patient selection. These interventions play a significant role in reducing the issues associated with opioid prescription.^41^ Cost-saving interventions, such as substituting dosage forms or transitioning patients to outpatient services, also contribute to better patient health and satisfaction outcomes.^42,43^ Only a small proportion (0.6%) of interventions made by clinical pharmacists are suboptimal, but they can negatively impact therapeutic practices and should be minimized to promote good prescribing practices.^44^

This study evaluated the clinical pharmacist interventions primarily involving opioids and patients with cancer in Pakistan, where research on these medications and populations is limited. It sets the stage for further exploration of opioid medications and OSP in the country.^45^ As controlled substances graded by law, opioids are kept under lock and key in an area inaccessible to the public.

This mono-centric retrospective study was conducted exclusively in an oncology setting, focusing on a specific number of patients and clinical pharmacist interventions. generalizability of the results may be enhanced by designing a multicenter prospective study in non-cancer settings to increase sample size, provide a more comprehensive assessment of clinical pharmacists’ role in opioid use, and highlight the significance of OSP in Pakistan. Additionally, because it was a retrospective study, assessing patient pain, function, or other measures was impossible, limiting a complete understanding of intervention effects. The focus on pharmacist interventions within medication orders for patients with cancer precluded a comprehensive demographic assessment. Data collection primarily involved analyzing prescribing issues rather than demographic specifics, thus limiting the inclusion of total patient numbers. Due to the retrospective nature and time constraints, physicians were not surveyed to assess the factors behind their acceptance of pharmacist interventions.

## Conclusion

In conclusion, our study represents a significant contribution to understanding the role of clinical pharmacists in addressing opioid prescribing issues, particularly in oncology care in Pakistan. The high rate of pharmacist interventions and substantial acceptance by physicians highlight the effectiveness of integrating pharmacists into interdisciplinary healthcare teams. These interventions not only rectify prescribing issues but also optimize opioid therapy, reducing the risk of adverse events and improving patient outcomes. Our findings underscore the need for comprehensive stewardship programs to minimize prescription issues and enhance the quality of opioid prescribing practices. While valuable, future research should explore the broader implementation of opioid stewardship programs across various healthcare settings to enhance patient care and safety further.
